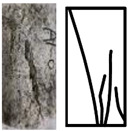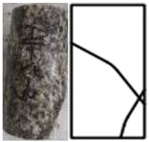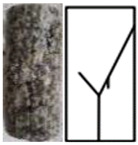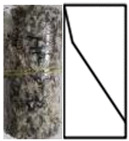# Evolutionary Analysis of Heterogeneous Granite Microcracks Based on Digital Image Processing in Grain-Block Model

**DOI:** 10.3390/ma15051941

**Published:** 2022-03-05

**Authors:** Guanlin Liu, Youliang Chen, Xi Du, Suran Wang, Tomás Manuel Fernández-Steeger

**Affiliations:** 1 Department of Civil Engineering, School of Environment and Architecture, University of Shanghai for Science and Technology, Shanghai 200093, China; 181520111@st.usst.edu.cn (G.L.); xi.du@unsw.edu.au (X.D.); evanwangsuran@foxmail.com (S.W.); 2 Institut für Angewandte Geowissenschaften, Technische Universität Berlin, Ernst-Reuter-Platz 1, BH 3-1, 10587 Berlin, Germany; fernandez-steeger@tu-berlin.de

**Keywords:** heterogeneous rock, microcrack evolution, digital image processing, damage mechanism, grain-block model

## Abstract

Rocks are natural materials with a heterogeneous microstructure, and the heterogeneity of the microstructure plays a crucial role in the evolution of microcracks during the compression process. A numerical model of a rock with a heterogeneous structure under compression is developed by digital image processing techniques and the discrete element method. On the grain scale, the damage mechanism and microcrack characteristics of a heterogeneous Biotite granite under compression fracture are investigated. First, the process of constructing a digital image-based heterogeneous grain model is described. The microscopic characteristics of geometric heterogeneity, elastic heterogeneity, and contact heterogeneity are all considered in the numerical model. Then, the model is calibrated according to the macroscopic properties of biotite granite obtained in the laboratory, and the numerically simulated microcrack cracking processes and damage modes are obtained with a high degree of agreement compared to the experiments. Numerical simulations have shown the following: (1) Microcracking occurs first at the weak side of the grain boundaries, and the appearance of intragranular shear cracks indicates that the rock has reached its peak strength. (2) The stress concentration caused by the heterogeneity of the microstructure is an essential factor that causes rock cracks and induces rupture. Intragranular cracks occur successively in quartz, feldspar (plagioclase), and biotite, with far more intragranular cracks in quartz and feldspar (plagioclase) than in biotite. (3) Microcracking in quartz occurs as clusters, fork and fracture features, and in feldspar (plagioclase) it tends to cause penetration microcracking, which usually surrounds or terminates at the biotite. (4) As the confining pressure increases, the tensile break between the grains is suppressed and the number of shear cracks increases. At the macro level, the rock failure mode of the numerical model changes from split damage to shear destruction, which is consistent with the law shown in laboratory experiments.

## 1. Introduction

The deformation of rocks during compression is mainly caused by the closure, propagation, coalescence and eventual formation of macroscopic cracks in internal microfractures [1,2,3,4,5,6]. The evolutionary process of these microcracks plays a decisive role in the stability of the overall rock structure. However, rocks are naturally heterogeneous materials, and even the same rock has widely varying crack morphology and compressive strength values. Many uncertain factors contribute to this large variability, such as mineral composition, microscopic inhomogeneity, degree of erosion, and degree of weathering [7]. Therefore, many researchers have studied the strength of granites in terms of weathering [8,9], mineral weakening [10], mineral composition [11], grain structure [12], grain size [7,13], etc. The above studies show that the complex macroscopic reflections of rocks are mainly constructed by the intrinsic micromechanical behavior, which is dominated by mineral heterogeneity and strongly influences fracture stress and peak strength. Lan et al. [14] described microstructural heterogeneity as three aspects: (a) geometric heterogeneity refers to changes caused by variations in grain size and shape; (b) elastic heterogeneity refers to elastic changes caused by variations in grain stiffness of different components; and (c) contact heterogeneity refers to contact changes caused by anisotropy in the contact distribution (length and orientation) and stiffness anisotropy between grains. During indoor rock compression tests, heterogeneous stress fields are generated within the specimen due to microscopic heterogeneity between constituent grains and the presence of their own microdefects (pores, uncracking, and grain boundary voids) [14,15,16,17,18]. As the stress increases, the local tensile strength near the defects or at the grain boundaries exceeds its own tensile strength, and new microcracks are generated [17,18,19,20]. With increasing loads, microcracks continue to grow and merge, eventually leading to macroscopic damage in the form of axial splitting (under unconfined conditions) or oblique shear brand (under high confinement) [21,22]. It can be seen that rock fracture damage evolution mainly depends on the complex interaction between microcracks and heterogeneous stress fields.

In the past few decades, numerous scholars have attempted to investigate the behavior of microcracking evolution during the compressional rupture of brittle rocks under heterogeneous conditions utilizing various advanced monitoring techniques. For example, the transient elastic waves generated by crack breakage are monitored through acoustic emission (AE), which indirectly records the crack development during the loading process of the rock [23,24]. Scanning electron microscopy (SEM) allows direct observation of the apparent characteristics of microcracks, making it possible to observe cracks during the damage process and providing a basis for quantitative analysis of the microcrack evolution process [16,25,26]. With technological advances, X-ray computed tomography (CT) is used to observe damage caused by microcracks in rock specimens [27,28]. Nishiyama and Kusuda [27] proposed a fluorescence method that allows direct observation of the density and type of microcracks produced within a rock sample. This method was also used to study crack initiation and propagation phenomena under different loading conditions [28,29]. Although the experimental methods described above have been widely used to study microfracture behaviour and stress damage in rocks, the mechanism of the damage process remains unknown due to the heterogeneous nature of rocks. The use of numerical simulation methods can provide a good complement to indoor experiments, especially when the effect of microstructural heterogeneity on cracking needs to be considered. Therefore, grain-scale numerical models that can take into account the microstructural level are gaining more and more attention [14].

The grain-scale modeling method belongs to the Discrete Element Method (DEM), and the common modeling types are circular particles and polygonal blocks, which essentially represent a collection of basic units that are cohesive and free to deform, displace and rotate [30,31]. These two modeling approaches are widely accepted for four important factors: 1. The ability to create model grains with similar shapes to the actual mineral particles; 2. The ability to consider numerous micro heterogeneous aspects (component heterogeneity, contact heterogeneity); 3. The ability of the modeling approach to simulate the natural crack formation and cracking; 4. The ability to continuously monitor macroscopic and microscopic data during the rock fracture process.

Circular particle modeling is called bonded particle model (BPM) in the DEM method and is mainly run in Particle Flow Code (PFC) [32]. In the BPM, the rock specimen consists of round particles bonded by parallel bonding and linear contact law interactions. However, BPM has a limitation in that the ratio of tensile to compressive strength in the model is much higher than that measured in experiments [33]. To solve this problem, Potyondy [34] proposed the grain-based model in PFC (PFC-GBM), in which rocks are represented as polygonal particles glued along their adjacent edges, each polygonal particle consisting of many small disc particles. This method not only solves the problem of the tension and compression ratio, but also allows the user to set different particle sizes of the minerals. This polygonal grain structure enables a more realistic reproduction of the microstructure in the rock material.

Another more popular DEM is the block element approach, which is usually represented by the Universal Discrete Element Code (UDEC) [35]. Similar to the circular particle construction process, this method involves bonding the basic block grains to form a complete rock sample. The difference is that the individual block is polygonal with four or more sides. Since the polygonal blocks are random in shape and size, a better interlocking effect can be produced between the blocks [14,36]. Polygon blocks usually are generated using the Voronoi tessellation technique, which employs a set of seeds to divide the space into polygonal cells [14]. This grain model with Voronoi polygon block in UDEC (UDEC-GBM) represents the petrographic grain characteristics in crystalline rocks [14,37]. This model can also be used to define the microscopic heterogeneity of reduced rocks with different grain sizes and crystal content. At the grain scale, both PFC-GBMs and UDEC-GBMs are sufficient to establish microstructural heterogeneity and realistically replicate the rock fracturing process [38,39,40]. However, UDEC-GBMs have the shortcoming of not allowing the generation of transgranular fracturing within the grain itself and the inability to characterize the true texture structure on the grain [41]. Although PFC-GBM can simulate intracrystalline damage, the circular shape of the grains results in higher porosity, making the ratio of tensile strength to uniaxial compressive strength (UCS) too high, which results in low friction strength in the macroscopic experiment [33,41].

In this study, digital image processing was applied to the microstructure of the grain-based model to investigate the damage mechanism and microcrack evolution characteristics of Biotite granite. This method has advantages in studying the heterogeneity of rock microstructure and has been widely demonstrated [42,43,44]. First, the grain-based UDEC-Voronoi model is introduced, setting the size of the base block to 1/3 of the actual grain size, which means that each natural size grain consists of 2–3 numerical sub-blocks and, therefore, allows the construction of cross-grain cracking effects. A numerical model of the granite consistent with the microstructure was established by digital image processing, taking into account not only the heterogeneity of microscopic composition distribution but also the contact heterogeneity between different grains. The calibration process is based on the results of laboratory tests on Biotite granite specimens. The microcrack evolution patterns and microcrack patterns among different minerals under uniaxial compression were investigated in detail to reveal the damage mechanisms at the microscopic level. The damage evolution and damage patterns of the model under various loading conditions were analyzed and discussed by comparing them with the indoor tests.

## 2. Grain-Based Model Method in UDEC

**Figure 1:**
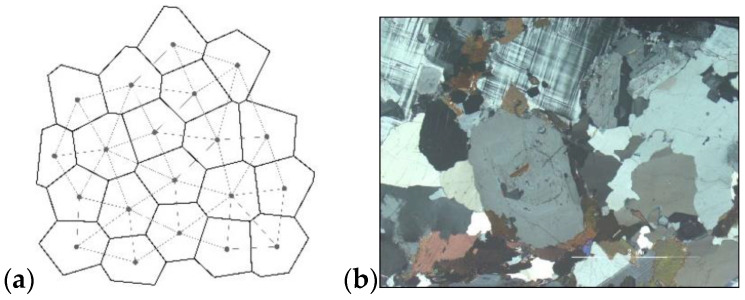
(**a**) Voronoi grain-model (**b**) Natural mineral grain structure in rock.

The Voronoi mosaic framework has been verified to be naturally consistent with the granular mesoscopic or microscopic structure of the rock material [14,44]. UDEC has an in-built Voronoi model which is assemblage of distinct deformable polygons; the model generation process follow these steps [45]: (1) Random points, (2) Generation of Delaunay triangulation, (3) Generation of Voronoi tessellation, (4) Generation of Voronoi model in UDEC. The final Voronoi grain model is similar to the natural mineral grain structure (See Figure 1). 

The mechanical properties of the connection between Voronoi polygon blocks (representing both inter and intra grain bonds) obey the Coulomb friction law.

In the normal direction, the contact force-displacement relation is assumed to be linear and governed by its normal stiffness $\left(k_{n}\right)$ such that

(1) $$\Delta \sigma_{n}=-k_{n}\Delta u_{n}$$

where $\Delta \sigma_{n}$ is effective normal force increment and $\Delta u_{n}$ is the normal displacement increment. A limiting tensile strength, $\sigma_{n}^{max}$, is assumed for any contact. If the tensile strength is exceeded $\left(\sigma_{n}\leq -\sigma_{n}^{max}\right)$, then $\sigma_{n}=0$, and the contact is marked as a tensile crack. 

In the shear direction, the response is controlled by contact shear stiffness $\left(k_{s}\right)$. The shear stress ($\tau_{s}$) is limited by a combination of the cohesion $\left(C^{cont}\right)$ and friction $\left(\varphi^{cont}\right)$. Thus, if

(2) $$\left|\tau_{s}\right|\leq C^{cont}a_{c}+\sigma_{n}tan\varphi^{cont}=\tau_{s}^{max}$$

then,

(3) $$\Delta \tau_{s}=-k_{s}\Delta u_{s}^{e}$$

or else, if

(4) $$\left|\tau_{s}\right|\;\geq \;\tau_{s}^{max}$$

then

(5) $$\tau_{s}=sign\left(\Delta u_{s}\right)\;\tau_{s}^{max}$$

where $a_{c}$ is contact areas, $\Delta u_{s}^{e}$ is the elastic component of the incremental shear displacement and $\Delta u_{s}$ is the total incremental shear displacement. The contact is marked as a shear crack if Equation (4) is achieved.

**Figure 2:**
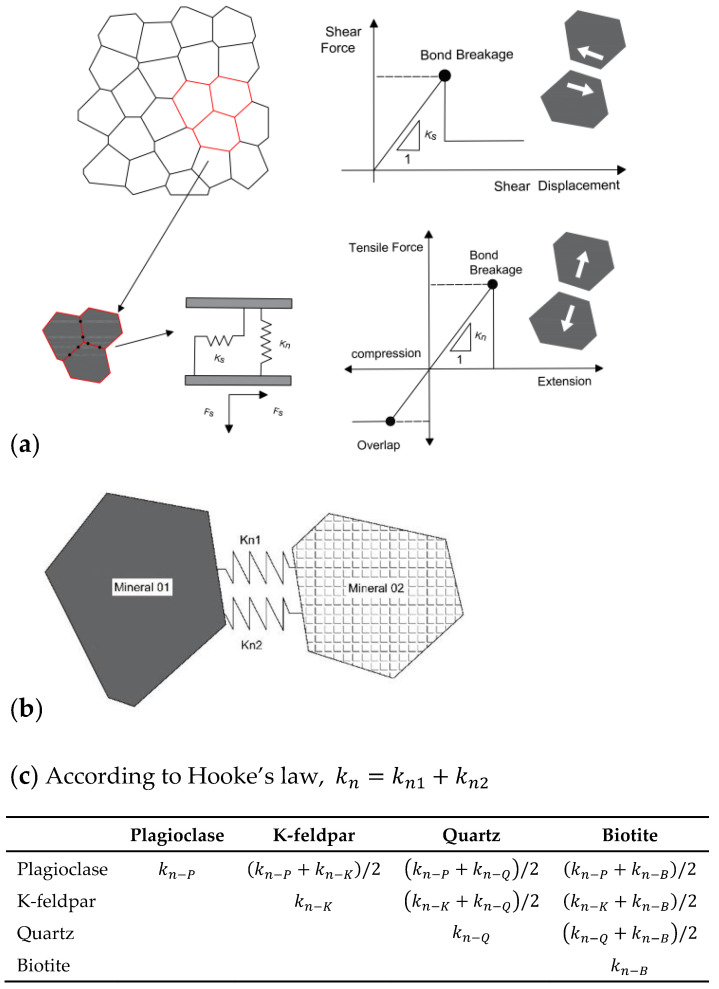
Constitutive behavior of Voronoi grain-model. (**a**) Joint connection mode and fracture criterion, (**b**) Normal stiffness connection between different grains in rock model (modified from [46,47]), (**c**) Calculation table of equivalent stiffness between different grains. Note: $k_{n}$ is equivalent stiffness, $k_{n1}$ and $k_{n2}$ is stiffness of two adjacent blocks.

After one contact breaks, forces are redistributed, and it might cause adjacent contacts to break. There is no need for a complex constitutive model to control cracking behavior during the process [37]. Figure 2 illustrates the contact normal and shear behavior between different grains in the rock model.

## 3. Model Description and Calibration

**Figure 3:**
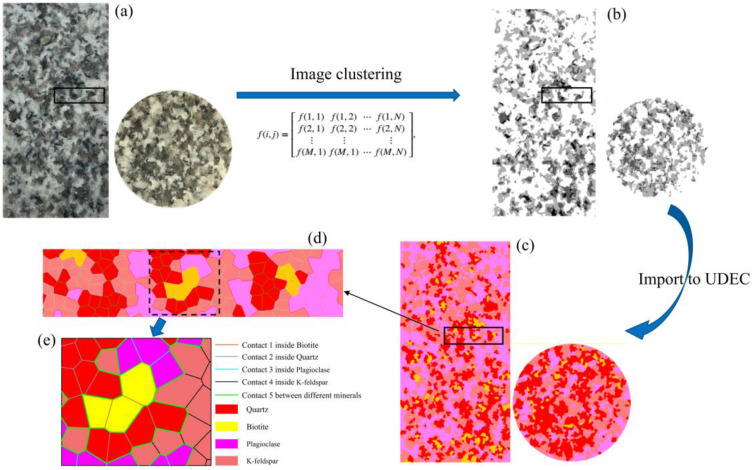
Heterogeneous rock numerical modeling process based on digital image processing. (**a**) Digital images of test samples (Uniaxial/Triaxial and split test samples); (**b**) Cluster image of different crystal components; (**c**) Numerical Modeling of Heterogeneity; (**d**) Partially magnified image; (**e**) Joint connection heterogeneity of different crystal compositions.

In the microscopic view, rocks are constitutionally heterogeneous materials, consisting of grain blocks of different mineral components crystallized and cemented together. Differences in composition, distribution, grain morphology and grain size of multiple minerals can significantly affect the strength and fracture mechanism of rocks. The rock sample in this study is a biotite granite from Changsha, Hunan, China. To investigate the evolution of cracks during the compression of the rock in a more realistic way, the effect of heterogeneity is considered in this paper. X-ray diffraction analysis of this rock sample showed that the main components of the sample consisted of plagioclase (36.2%), potassium feldspar (29.1%), quartz (27.8%), and biotite (5.7%), in addition to kaolinite (0.2%) and a small number of other materials (<1%) (see Table 1). The grain sizes of plagioclase, K-feldspar and quartz are primarily in the range of 2–5 mm, and the grain sizes of biotite are in the range of 0.5–3 mm, as obtained from the microscopic examination of the thin sections of samples (see Table 2). Therefore, the main four mineral compositions of plagioclase, K-feldspar, quartz and biotite are set in the corresponding models (see Table 2). The average size of the model grain is set according to the natural crystal grain size. The numerical image processing technique is used to restore the distribution position and properties of different grain components inside the rock to avoid the influence of different component distribution positions on the process of fracture under pressure. The Voronoi grain block generated by the above method has the advantage of similar morphology to the grain blocks and further restores the crystal structure details. The final heterogeneous grain-block model with a microstructure similar to that of the rock sample is generated, as shown in Figure 3.

### 3.1. Numerical Specimen Model Setup Based on Digital Image Processing

Digital image processing (DIP) is a computerized technique that electronically captures a scene and converts it into a two-dimensional pixel image, then extracts important information through mathematical algorithms. The use of DIP can effectively obtain the surface microstructure of rock samples [42,43,44]. Its basic principle is to distinguish different components and their locations based on the brightness of varying mineral colors in the digital images of rocks. The granite studied in this paper is mainly composed of four different colors of quartz, k-feldspar, biotite and plagioclase. Therefore, this method can effectively distinguish the composition structure of granite. However, the accuracy of the differentiation will directly affect the numerical model micro-composition, so the key issue of rock image DIP is how to make accurate segmentation between different minerals. The existing image segmentation methods are mainly divided into the following categories: threshold-based segmentation methods, region-based segmentation methods, edge-based segmentation methods and segmentation methods based on specific theories, etc. [48]. One of the simplest and most efficient methods is clustering, which can aggregate and classify specified objects into different clusters [49]. The most commonly used clustering method is the K-means clustering algorithm. The advantage of the K-means algorithm is that it is an unsupervised algorithm and does not need to manually set any thresholds [50]. Therefore, the use of this method excludes the dependence of the results on the subjectivity of the threshold selection and is computationally more efficient.

Firstly, the digital image of the granite specimen section is transformed into a grayscale image, where the image brightness is perceived at each pixel and assigned an integer value called gray level (Figure 3a,b). The integer value of gray level (value) ranges from 0 to 255. When the image tends to be black, the corresponding pixel value tends to 0; otherwise, the value tends to 255. The commonly used pixel matrix of grayscale images is:

(6) $$f\left(i,j\right)=\left[\begin{array}{ccc} f\left(1,1\right) & f\left(1,2\right) & \begin{array}{cc} \cdots & f\left(1,N\right) \end{array}\\ f\left(2,1\right) & f\left(2,2\right) & \begin{array}{cc} \cdots & f\left(2,N\right) \end{array}\\ \begin{array}{c} \vdots \\ f\left(M,1\right) \end{array} & \begin{array}{c} \vdots \\ f\left(M,1\right) \end{array} & \begin{array}{cc} \begin{array}{c} \cdots \end{array} & \begin{array}{c} \vdots \\ f\left(M,N\right) \end{array} \end{array} \end{array}\right]$$

where *f* is the gray value; *i*, *j* represent the pixels corresponding to the rows and columns; and *M*, *N* is the total number of rows and columns of pixels, respectively. The numbers of the pixels along the *i*-axis and the *j*-axis are 400 and 200, respectively. The actual size of the image is 100 × 50 mm. Therefore, each pixel represents 0.25 mm.

**Figure 4:**
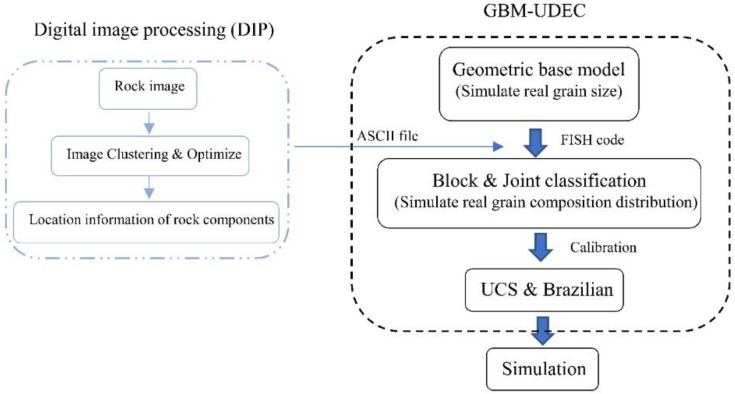
Flow chart of numerical model establishment and correction based on digital image processing.

Then, the K-means clustering algorithm was used to cluster the numerous integer values of gray levels, and the final four crystal components were clustered to match the actual ones, namely: plagioclase with the highest brightness (high gray level), potassium feldspar with the second-highest brightness (medium to high gray level), quartz with the second-lowest brightness (medium to low gray level), and biotite with the lowest brightness (low gray level). Subsequently, the image details were optimized and the coordinates of the pixel locations corresponding to the different components were extracted (Figure 3b). All the coordinate information was exported as an ASCII file, which could then be imported into the UDEC program via the UDEC internal FISH language (Figure 3b–d). The final numerical model of the same microstructure was created in UDEC’s GBM model and the flow chart is shown in Figure 4. Although digital image processing has largely restored the microscopic heterogeneity of the rock samples, some details are still ignored compared with the actual microstructure, and the main microstructure is mainly retained.

### 3.2. Numerical Specimen Model Setup

**Figure 5:**
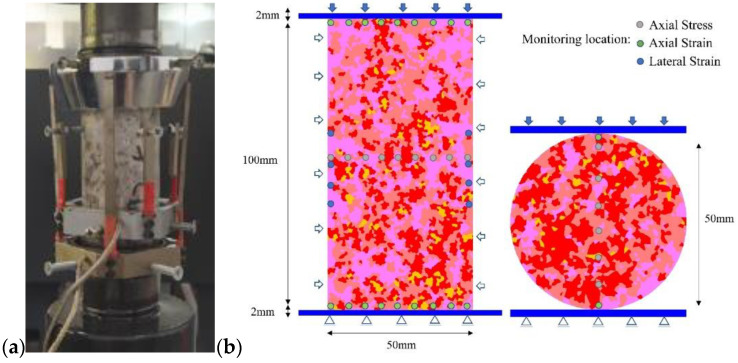
Boundary conditions of numerical model and layout of monitoring points. (**a**) Experimental sample monitoring system layout. (**b**) Layout of detection points for numerical simulation.

The test samples and standards followed ISRM [51], the numerical models of Uniaxial/Triaxial compression and Brazilian tension tests were established, as shown in Figure 5. The grain block is assigned as elasticity. The connection between the blocks follows the Coulomb slip model with residual strength properties [47].

The grain block size in the numerical model also has a significant influence on the failure mode [37,45,52]. The effect of size can only be ignored if the block size is less than 1/10 of the minimum model sample size [45,47]. Therefore, the average grain size of the model was set to 1/3 of the actual grain size, approximately 0.75 mm. This ensures that each natural size crystal grain is composed of 2–3 numerical sub-blocks (see Figure 2a), which can be regarded as the transgranular cracking effect of grain blocks in the process of compression and reduces the influence of grain size. The locations of monitoring points set in the numerical model are arranged according to the strain gauge monitoring method in the experiment (Figure 5a). The bottom of the model is fixed in both horizontal and vertical directions. The loading rate is set in the vertical direction of the top, while the horizontal direction of the top remains fixed. The loading rate is set to maintain the displacement rate at 0.01 m/s in both compression and tension tests, which is proved to ensure the model remains in a quasi-static condition [37,45]. 

### 3.3. Calibration Procedure and Results Analysis

In UDEC-GBM, the rock material is considered a combination of glued structural units. Its mechanical behavior is controlled by the contact of microscopic parameters between grain blocks, which cannot be set directly from experiments. Therefore, the microscopic parameters need to be corrected to match the macroscopic physical properties before the numerical simulation, to ensure the validity and accuracy of the numerical model in the calculation. This is a very critical step before the numerical simulation.

Many studies have confirmed a certain connection between the selection of microscopic parameters and macroscopic physical quantities [37,45,53]. By matching the simulated macroscopic response with the macroscopic properties such as Young’s modulus, Poisson’s ratio, uniaxial compressive strength, and tensile strength obtained from indoor experiments, accurate microscopic parameters are finally obtained. The process of iterative trial-and-error and calibration of the model adheres to the procedure outlined by Kazerani and Zhao [37], Stavrou [46] and Gao [54] by taking the following steps:

1. Calibration—Step 1: Macrophysical parameter setting

The microscopic grain block properties (density, shear modulus and bulk modulus) of the four components plagioclase, K-feldspar, quartz and biotite are their own physical properties. Therefore, they can be set directly by the data obtained from the macroscopic experiments. The normal and shear stiffnesses of grain block contact are derived from equation [35]:

(7) $$k_{n}\left(\;k_{s}\right)=10\left[\frac{K+\frac{4}{3}G}{\Delta z_{min}}\right]\;,\;n=1\sim 10$$

Δ*Z_min_* is the smallest width of zone adjoining the contact in the normal direction. 

The stiffness of the contact is determined by the connection parameters of the adjacent grain types, followed by Hooke’s law, see Table 3 and Figure 2b.

2. Calibration—Step 2: Deformation calibration

The normal to shear stiffness ratio determines the variation of Poisson’s ratio. The stiffness ratio of the intracrystalline contact is adjusted first to achieve the correct Poisson’s ratio of the mineral particles, and then the stiffness of the intergranular connection is adjusted to match the Poisson’s ratio of the macroscopic rock sample. Once the contact stiffness ratio was set, both the normal stiffness and block deformability were altered to fit the macro-Young’s Modulus.

3. Calibration—Step 3: Strength calibration

Through triaxial compression and Brazilian disk numerical simulation experiments, the intracrystalline contact of the joint friction angle ($\varphi_{j\;}$), joint cohesion ($c_{j}$) and joint residual friction angle ($\varphi_{jr}$) were first adjusted to correct the strength values of mineral grains; then, the inter-grain strength parameters were adjusted to match the strength values of macroscopic rock samples (uniaxial compressive strength, tensile strength, cohesive force, and internal friction angle.

Based on the above calibration process, a series of numerical simulations are performed to determine the values of intergranular and intracrystalline junction microscopic parameters. Finally, the calibrated suite of parameters of the GBM model is summarized in Table 3. 

**Figure 6:**
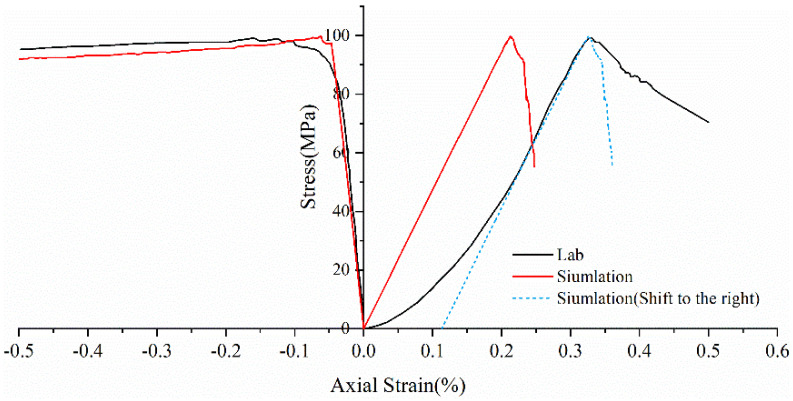
Comparison of stress–strain curves between numerical simulation and experiment (the blue dotted line is the comparison curve).

**Figure 7:**
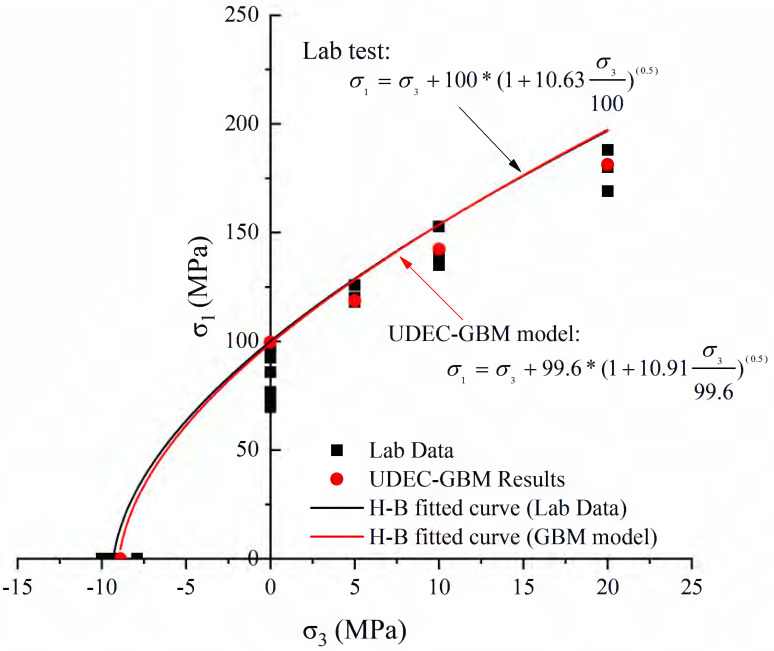
Hoek–Brown curve was compared between simulated strength curve and laboratory data.

**Figure 8:**
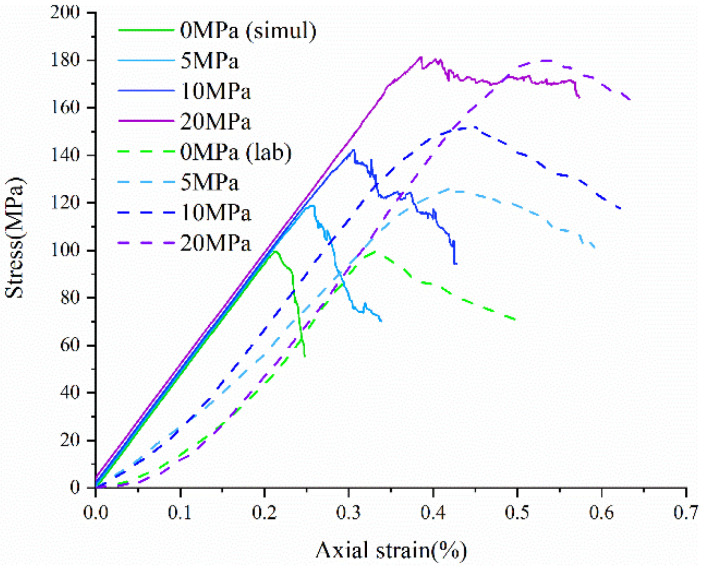
Comparison of numerical simulation and experimental stress–strain curves under different confining pressure conditions.

**Figure 9:**
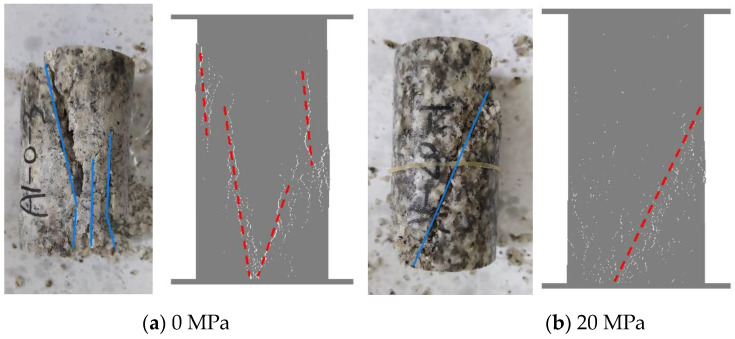
Comparison of the failure mode between the laboratory tests and the simulated ones. (**a**) 0 MPa. (**b**) 20 MPa.

Real rock samples have original cracks and pores, so the stress–strain curves in the laboratory have a compaction stage in the early stage, while the original cracks and pores are ignored in the numerical simulation of the Voronoi model, thus making the numerical simulation skip the crack closure phase and go directly from linear elasticity to elastic-plasticity to plastic yielding. Therefore, the model correction needs to remove the nonlinear phase (crack compacting stage) at the beginning of the experiment. The stress–strain curve of the corrected model can be obtained in agreement with the experimental results (blue dashed line) shown in Figure 6. Table 4 compares the macroscopic mechanical properties of granite obtained from experiments and numerical simulations, and the error values of macroscopic parameters obtained from simulated specimens are less than ±7%. Figure 7 compares the failure envelopes of the experimental and UDEC-GBM numerical simulations. These two comparisons show that both the simulated numerical strength and deformation characteristics before peak strength are in good agreement with the experimental results (See Figure 8). In addition, the simulated damage patterns are similar to the laboratory results, as shown in Figure 9. Damage to the rock specimens in the uniaxial compression test was dominated by axial splitting and shear coupled-mode damage. However, the specimens were mainly damaged in shear mode at a confining pressure of 20 MPa. The results obtained from numerical simulations have a certain degree of variability with experimental results; for example, the compaction stage cannot be considered and the numerical curves start from the elastic phase. The numerical simulation results of the post-peak curves under different surrounding pressures are slightly different from the indoor test results. However, the main mechanical behaviors of the rock specimens are well reflected from the overall point of view. Therefore, the present numerical model can be used for both macroscopic and microscopic analyses.

## 4. Results and Discussion

### 4.1. Stress–Strain Curve and Crack Threshold Analysis

**Figure 10:**
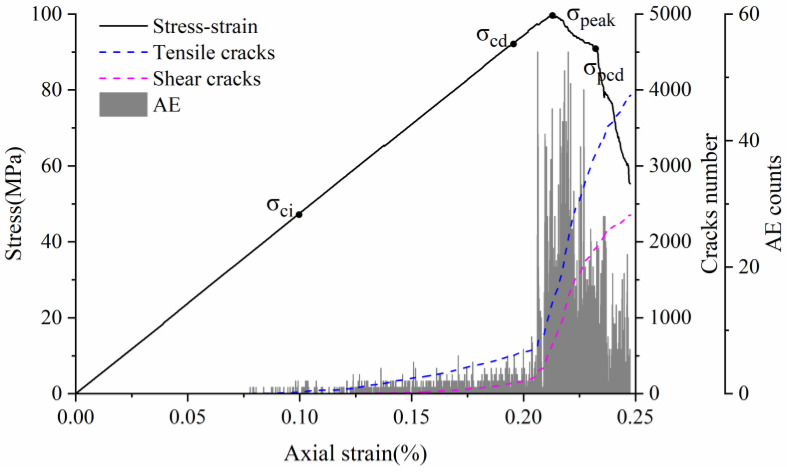
Stress–strain and AE curves of the numerical model rock.

Eberhardt et al. [5] performed a series of unconfined compression tests on brittle rocks and found that two main thresholds exist before peak strength is reached: (1) crack initiation stress threshold (σ_ci_) is determined by the first appearance of the crack and the beginning of the stable extension of the crack; (2) crack damage stress threshold (σ_cd_) is determined by the unstable development of cracks, mainly in the form of a sharp increase in the number of cracks starting or a reversal of the volume strain change. Both thresholds are identified by acoustic emission and strain monitoring. There is a gap between the numerical simulation method and acoustic emission detection in reality. However, acoustic emission characteristics can be approximated by detecting the stress state of joints and judging and recording the crack generation. This acoustic emission simulation method has been well used in previous studies [49,55,56]. Because the UDEC-GBM model can effectively determine the state of the connection between granular blocks, it can monitor and record the whole process of crack development and has natural advantages in terms of statistics on cracks. Therefore, the change in the number of cracks can be used to determine the above two thresholds. The complete stress–strain curves, crack evolution and number of acoustic emission events obtained from the simulations are shown in Figure 10. 

For most rocks, the crack initiation threshold is usually between 1/3 and 2/3 of the peak strength [3,57], and the crack damage threshold is usually between 70% and 90% of the peak strength [58]. As can be seen from Figure 10, when the strain is 0.1%, cracks begin to appear and grow at a stable speed (AE variation is constant), so the Crack Initiation (CI) stress threshold is defined here. At this point, the only form of cracking is tensile cracking, corresponding to axial stress of 43 MPa, 45% of the uniaxial compression strength. When the strain exceeds 0.19%, the cracks tend to grow dramatically and the amount of change in AE appears to increase steeply. This point is related to the crack damage (CD) stress threshold, which is a parameter representing the stress level where crack coalescence starts. At this point, the form of cracking is the simultaneous accelerated growth of tensile and shear cracks, corresponding to axial stress of 89 MPa, which is 90.75% of the uniaxial compressive strength. In summary, the numerically simulated CI and CD generally agree with the range of experimental statistics, indicating that the model can effectively express the testing process. 

### 4.2. Transmission Mechanism Analysis of Microcracks in Different Failure Stages 

Generally, the crack development in the process of rock compression can be divided into five stages [59]:

- Per-peak stage
  Stage I: crack consolidation;
  Stage II: linear elastic deformation;
  Stage III: crack initiation and stable crack growth;
  Stage IV: crack damage and unstable crack growth.
- Post-peak stage
  Stage V: unstable crack growth with decreasing stress;
  Stage VI: unstable crack growth and failure of the rock specimen with decreasing stress.

**Figure 11:**
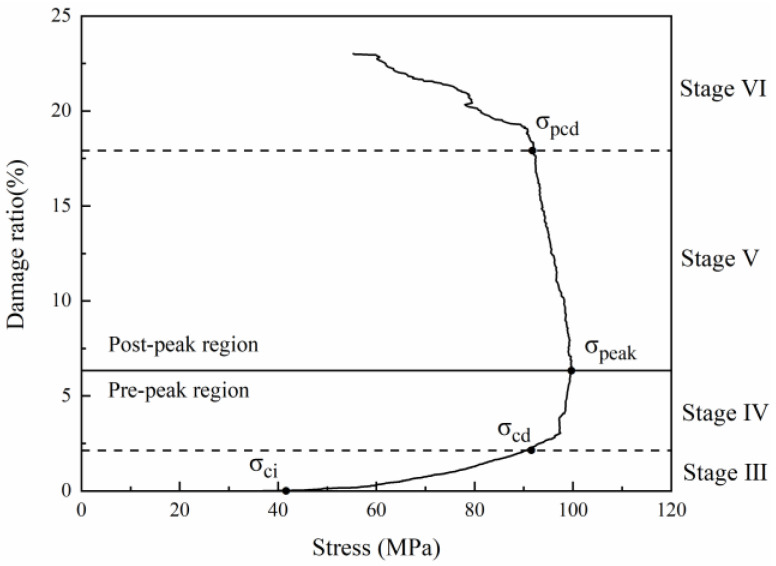
Cumulative damage and axial stress change curve. Note: Damage rate (%) = number of cracks/total number of joints × 100%.

The GBM-UDEC model did not consider the original cracks or pores in this study. Therefore, the crack closure stage is ignored, and the calculation starts from the elastic stage. Microcrack generation was investigated from Stage III to Stage VI, as shown in Figure 11.

#### 4.2.1. Boundary and Transgranular Crack Evolution Process Analysis

**Figure 12:**
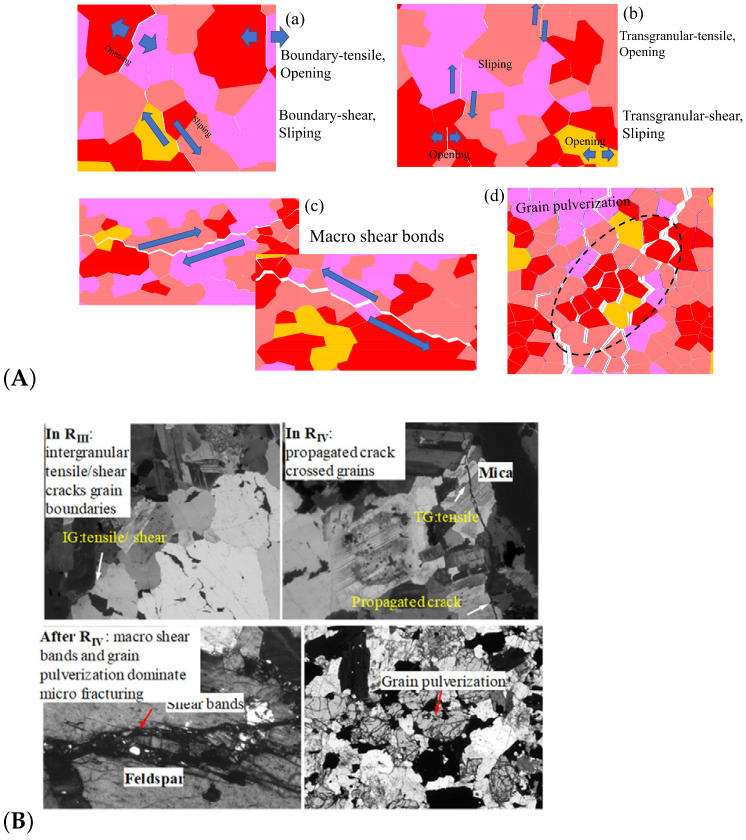
Typical micro-fracture characteristics during the four damage stages of Stage III, Stage IV, Stage V and VI: (**A**) Micro-fracture characteristics shown in numerical simulation. (**B**) Micro-fracture characteristics observed in experiments [7].

Since rock is a heterogeneous material, there is a great difference in the fracture pattern between grain blocks of different compositions during the compression process. The term ‘microcrack’ is classified as intragranular, transgranular, and intergranular cracks depending on the fracturing mechanism and the location where the cracks form [18]. Intergranular cracks are fractures that occur at grain boundaries. Intergranular and transgranular cracks are termed differently in the field of geology, with the former indicating cracks through one type of grain and the latter indicating cracks through multiple grains. In the present study, the underlying crystal particles of the model are not divisible as a whole, so this paper focuses on internal grain cracking damage of the same component and grain damage between different components. Grain contact damage within the same component is classified as transgranular crack, and grain contact damage between different components is seen as boundary crack, and thus can be classified into four types of microcracking according to the fracture forms: Boundary-tensile/shear cracks and transgranular-tensile/shear cracks. Figure 12a illustrates the development of these four types of cracks in the numerical model at different stages of damage in a manner consistent with the experimentally observed rupture of microcracks at different stages in Figure 12b.

**Figure 13:**
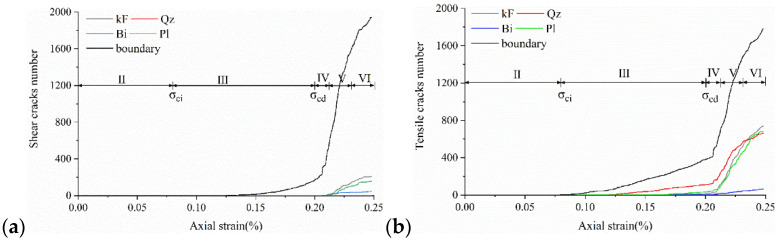
Variation curves of Boundary cracks and transgranular cracks at different damage stages: (**a**) The number of cracks produced by tensile failure. (**b**) The number of cracks produced by shear failure.

**Figure 14:**
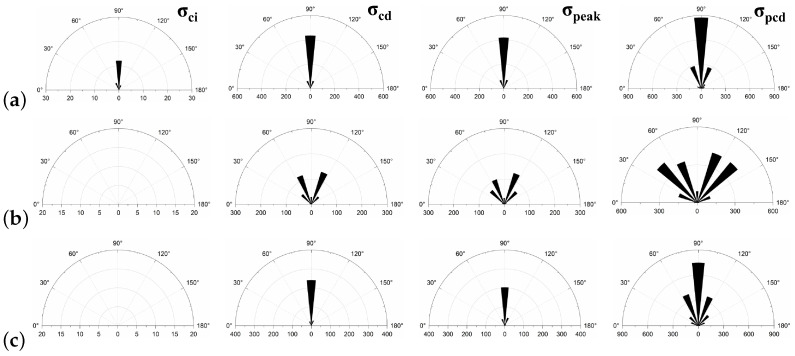

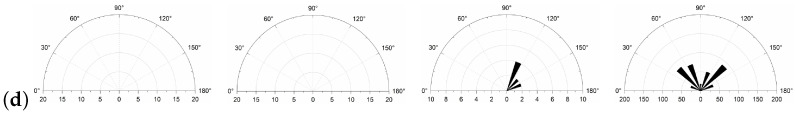
The crack number and angle statistics at the four stress values of σ_ci_, σ_cd_, σ_peak_ and σ_pcd_ during the crack evolution process: (**a**) the number of Boundary-tensile crack angles (**b**) the number of Boundary-shear crack angles (**c**) the number of Transgranular-tensile crack angles (**d**) the number of Transgranular-shear crack angles.

**Figure 15:**
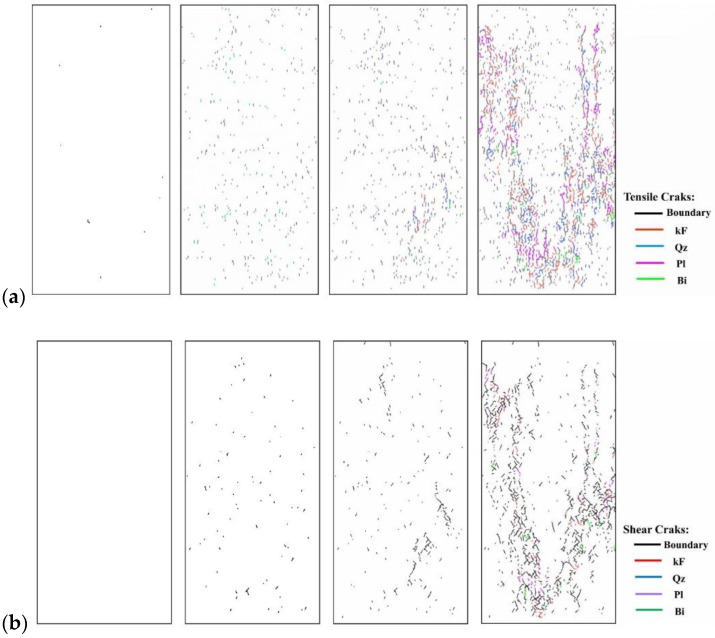
Crack distribution diagrams of transgranular cracks (kF, Qz, Pl, Bi) and Boundary cracks at four stress values of σ_ci_, σ_cd_, σ_peak_ and σ_pcd_ during the crack evolution. (**a**) Tensile failure (**b**) Shear failure.

To show in more detail, the variation curves of the number of grain boundaries and transgranular cracks at different stages of damage with strain under tensile/shear damage are counted in Figure 13. The angle, number and distribution characteristics of the microcracks corresponding to the four stress characteristic points, σ_ci_, σ_cd_, σ_peak_ and σ_pcd_, were selected for quantitative analysis according to the different fracture stages, see Figure 14 and Figure 15. When the stress reaches σ_ci_, boundary cracks appear first, the damage distribution of cracks is discrete and only tensile cracks on grain boundaries are produced. The angle of cracking is also mainly concentrated in the 90° direction, with small cracks and low distribution density, and no other cracks are produced.

As the load increases, when the stress reaches σ_cd_, the grain boundary crack density gradually increases, the fracture of the grain boundary is not in the form of a single tensile, shear damage also occurs at the same time, forming mixed tensile-shear damage in the grain boundary. At the same time, a certain amount of transgranular damage also begins to appear, but only exhibits transgranular tensile damage. When the peak stress point is reached, the increase in the number of cracks in the form of tensile damage decreases, while the increase in the number of cracks in the form of shear damage increases, and shear cracks in the grain only start to appear after the peak strength is reached. When the peak stress point is reached, the increase in the number of cracks in the form of tensile damage decreases, while the increase in the number of cracks in the form of shear damage increases, and shear cracks in the grain only start to appear after the peak strength is reached. In combination with Figure 14 and Figure 15, it can be seen that the trend of shear damage dominates the formation of the macroscopic crack dip. When loaded to the post-peak crack damage stress point, both grain boundaries and transgranular cracks appear to increase significantly, cracks penetrate and connect with each other and the rock loses its load-bearing capacity at an accelerated rate.

In summary, on the microscopic scale, the fracture of microcracks in uniaxial compression tests occurs preferentially on the weak side of crystal boundaries, with the appearance of intracrystalline shear cracks signaling that the rock has reached peak strength. Fracture of microcracks follows the following sequence of development: boundary-tensile (open) crack, boundary-shear (sliding) crack, transgranular-tensile crack, transgranular shear crack, macro-shear bond.

#### 4.2.2. Different Failure Stages with Grain-Scale Fracturing Characterization in Uniaxial Compression 

**Figure 16:**
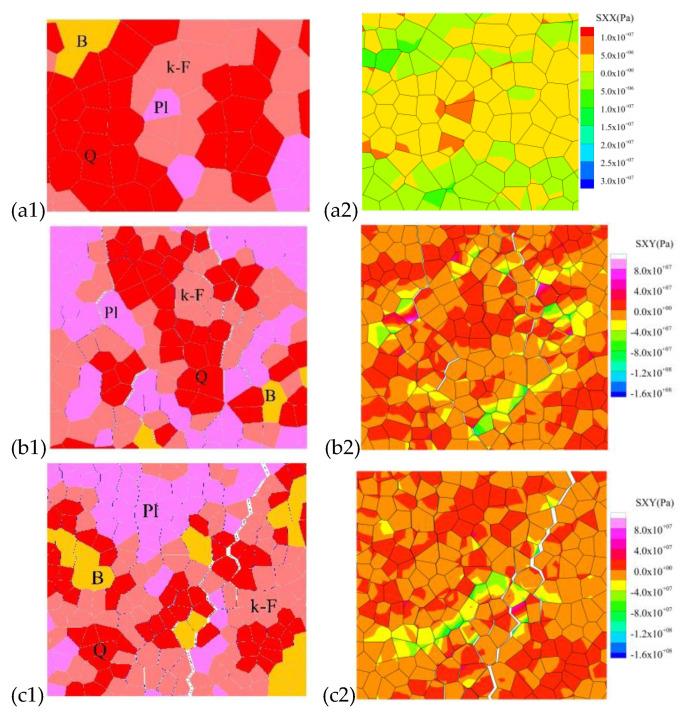
Local typical grain-scale block stress fracture characteristics and stress cloud diagram at different damage stages. (**a1**,**a2**) In Stage III, the grain boundary tensile failure stress cloud diagram; (**b1**,**b2**). In Stage IV, mixed intragranular the failure stress cloud diagram; (**c1**,**c2**). In Stage V and VI, the macroscopically penetrating failure stress cloud diagram.

**Figure 17:**
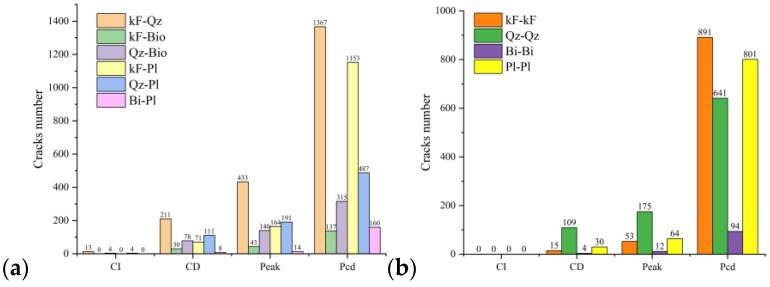
The crack characteristic statistics of Qz, kF, Pl, Bi at the four stress values of σ_ci_, σ_cd_, σ_peak_ and σ_pcd_ in the process of grain evolution. (**a**) Different crystals Boundary cracks between grains. (**b**) Transgranular cracks in the same grain composition.

In Stage III, initial cracking occurs mainly between the grain boundaries, mostly between quartz and other crystal particles (Qz-Pl, Qz-Bi, Qz-kF grain boundary cracks), and mostly at the tips of crystals (Figure 16a and Figure 17a). The main reason for this is that the quartz is the strongest among the three minerals, with a large modulus of elasticity and relatively small deformation during stress, which tends to form stress concentrations between the crystal boundaries of the different components. The bond strength at the grain boundary is much less than the bond strength within the crystal, the grain boundary cracks are the first to break down when the stress is concentrated, resulting in grain boundary fracture. Local values of the tensile stress count of the grain contact portion are shown in Figure 16. This is consistent with the findings obtained by Lan [14] and L.XF [41]. In addition to this, most grain blocks are polygonal, making it easier for stress concentrations to occur at the tip, thus causing further fracture of the grain boundaries. Biotite has a relatively low elasticity modulus and a high Poisson’s ratio. It can produce large elastic deformations during stressing, making it less likely to produce stress concentrations. Hence, the number of grain boundaries in contact with biotite produces the lowest amount of damage (Figure 17a).

In Stage IV, as the axial compressive stress increases and exceeds the crack damage stress, the extrusion between the grain boundaries becomes more severe, exceeding the bond strength value, and the fracture of the grain boundaries increases rapidly. The stress is transmitted from the weak part of the grain boundary to the internal skeleton (Figure 16b and Figure 17b), and the first tensile breakage occurs inside the quartz with the higher elasticity modulus, forming a through-grain tensile crack. Plagioclase and K-feldspar have similar elasticity modulus and both show the same rate of intracrystalline fracture. On the other hand, biotite has a lower elasticity modulus and a high Poisson’s ratio and does not fracture through the grain until near its peak strength. Grain boundary fracture is still the main form of fracture at this stage and a small amount of intracrystalline cracking is beginning to appear.

In Stages V and VI, after the peak strength is exceeded, the rock loses its main load-bearing capacity and cracks at the grain boundaries derive and join each other to form through macroscopic cracks. At this point, microscopic stress concentrations occur more often within the different components of the rock, with loads being carried mainly by grain blocks of the same composition (Figure 16c). Although grain boundary fracture still occupied the main failure mode at this stage, the stress heterogeneity was more concentrated in the intragranular; the situation of intragranular rupture increased rapidly, and even intragranular crushing occurred.

**Figure 18:**
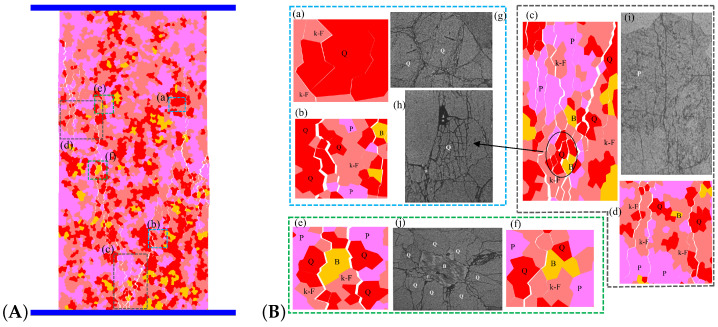
The fracture characteristics of micro-cracks between different rock components during uniaxial compression failure. (**A**) Macroscopic view of a rock with fabric heterogeneity during compression fracture; (**B**) Enlarged view of local cracking: subfigures (**a**–**f**), represented in numerical model. subfigures (**g**–**j**), represented observed in the experiment [60]. NOTE: The blue, black, and green colored dotted boxes represent the comparison of numerical simulation and experimental observation of quartz, feldspar, and mica when three crystals are fractured.

In order to compare the simulated failure mechanism of all microcracks (Figure 18A) with the observation results of the laboratory experiment (Figure 18B(g–j), modified from [49,60]), the selective amplification diagram as shown in Figure 18B is established. As can be seen in the enlarged view, the cracks in the quartz are often clustered in clusters (Figure 18B(a)), together with bifurcated cracks (Figure 18B(b)) and fractured areas with dense microcracking (Figure 18B(c), circled area in black). It is highly similar to the fracture form observed in experiments (Figure 18B(g,h)). The form of quartz fracture during the experiments was obtained by observing a large number of granite flakes. It was found that the original microcracks in quartz before loading were generally much more numerous than those in feldspar and biotite [61,62]. Thus, cracking in quartz is more likely to occur at lower stresses than in feldspar and biotite. However, crystal grains of the quartz component in the numerical model, which do not have original cracking, share a similar form of fracture, strongly indicating that stress concentration-induced fracture is also an essential factor in inducing quartz fracture.

It has been observed experimentally that there are usually one or two groups of joint planes in feldspar. These joint surfaces can easily expand into microcracks during loading [29,60]. It results in penetration cracks, as shown in Figure 18B(h), and this pattern is better reflected in the numerical model, as shown in Figure 18B(c,d).

Compared to quartz and feldspar, biotite has a relatively small modulus of elasticity, which significantly affects stress distribution and microcrack initiation and development (Figure 16 and Figure 18). Experimental studies have shown that microcracks will terminate in extension when they encounter biotite, or develop along with biotite, but rarely penetrate biotite unless the direction of the microcrack is parallel to its joints [29,44,60]. This shows that biotite is more ductile than quartz and feldspar, and it is less prone to microcracking. As a result, microcracks usually form or terminate around the biotite, forming crack ‘voids’, as shown in Figure 18B(j), and similar patterns have been found in numerical simulations (Figure 18B(e,f)).

### 4.3. Comparative Analysis of the Development and Evolution of Microscopic Grain Boundary Cracks and Intracrystalline Cracks under Different Confining Pressure Conditions

In practice, compressional damage in rocks is more likely to occur under the combined effect of different confining pressure conditions. Therefore, this paper further investigates the development and evolution of microscopic grain boundary fracture and intracrystalline fracture under different confining pressure conditions (0, 5, 10 and 20 MPa), and the results of the numerical simulations are tabulated in Table 5. Table 5 mainly shows the stress–strain relationships with grain boundary/transgranular crack evolution under different confining pressure conditions, the trend of tensile and shear fracture, as well as the statistics of the number and angle of cracks at sample damage. The results are elaborated and analyzed as follows.

#### 4.3.1. Uniaxial Compression

As the stress increases, both forms of cracking occur, with initial micro-cracks appearing in small numbers in a discrete manner, steady growth, agglomerative growth, and eventually converging into macro-cracks. Boundary tensile cracks are the first to appear at the crack initiation and show a steady growth trend. A few boundary shear cracks and intragranular tensile cracks begin to appear when crack damage is reached, at which point the number of boundary tensile cracks is approximately twice the sum of the number of boundary and intragranular shear cracks. Intragranular shear cracking only starts to occur when approaching the peak stress, and at this time, the number of boundary cracks is much higher than the number of intracrystalline cracks. The boundary strength parameter mainly causes this cracking phenomenon (i.e., joint cohesion and tensile strength) to be much lower than the intracrystalline strength parameter. The local stress concentration is mainly between the grain boundaries, thus causing microcracking. Tensile cracking has always been the dominant form of cracking in the evolution of cracking, with shear cracking occupying a secondary form. Grain boundary tensile cracks are approximately parallel to the axial (vertical) direction of loading (Table 5, 0 MPa), while grain boundary shear cracks and intragrystalline tensile cracks are usually about 45 degrees axially inclined. When the peak is reached, tensile cracks interact to produce secondary cracks and gradually develop into macroscopic cracks parallel to the direction of loading. However, shear cracks gradually develop into macroscopic diagonal cracks with axially inclined connections.

#### 4.3.2. Triaxial Compression

1. Confine pressure: 5 MPa

The initial cracking takes the form of grain boundary tensile cracking, which is consistent with crack generation in uniaxial compression mode, but the initial cracking requires higher stress conditions to occur and the rate of development of grain boundary tensile cracking is significantly reduced. When it reaches the crack damage stress threshold, the number of cracks produced at grain boundaries still dominates, with a small number of intracrystalline tensile cracks present. However, unlike the uniaxial compression case, grain boundary cracks are no longer dominated by tensile cracks, the number of grain boundary tensile cracks is equal to the number of grain boundary shear cracks and intra-grain shear cracks begin to appear. When the peak strength is exceeded, the interaction and combination of grain boundary microcracks and intracrystalline microcracks lead to the formation of macroscopic shear zones.

2. Confine pressure: 10 MPa

When the confining pressure is 10 MPa, the overall development of microcracking is dominated by grain boundary shear fracture. Initial cracks occur in the form of grain boundary tensile and shear cracks. Tensile cracks at grain boundaries develop more and more slowly, while shear cracks at grain boundaries develop rapidly. The main mechanism of crack initiation changes from shear to tensile. On the other hand, intracrystalline tensile and shear cracks increase rapidly as the peak strength is approached. Macroscopic cracking parallel to the loading direction gradually decreases and is dominated by inclined shear cracks.

3. Confine pressure: 20 MPa

The test results are similar to those at 10 MPa confining pressure, producing micro-cracks mainly in the form of grain boundary shear cracks, with the number of cracks far exceeding other forms of cracking. Grain boundary tensile cracking is restrained and inhibited by the higher confining pressure. Initial cracking occurs only in the form of grain boundary shear cracking. When the crack damage stress threshold is reached, a small amount of intracrystalline cracking occurs, with grain boundaries still dominate by shear cracking. When the peak stress is reached, boundary cracks and intracrystalline tensile cracks begin to show an increase, especially in the post-peak stage when both boundary cracks show a larger increase.

### 4.4. Comparative Analysis of Macroscopic Fracture under Different Confining Pressures

**Figure 19:**
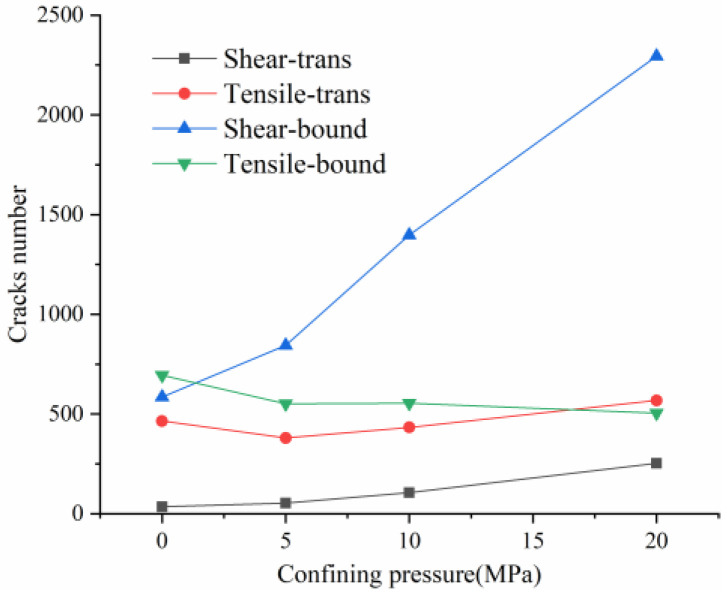
The quantitative variation of tensile/shear cracks on grain boundary and intracrystalline under different confining pressures at peak strength.

**Figure 20:**
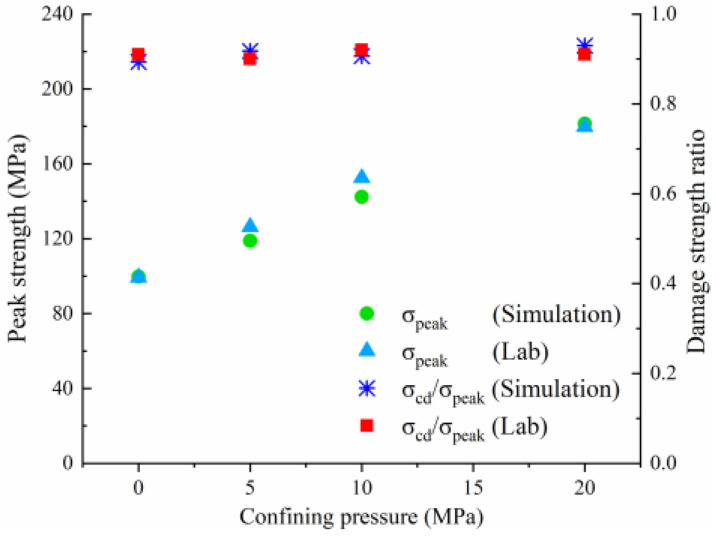
Relationship between peak strength and damage strength ratio under various.

Table 6 summarizes the macroscopic fracture of the numerical simulations and experiments for different confining pressures. Group A is a numerical simulation of the macroscopic damage model under different confining pressure conditions, which clearly shows a change from macroscopic through fracture to local shear damage as the confining pressure increases. In order to illustrate the damage pattern in more detail, the tensile and shear fracture zones of the model are reproduced in group B in the form of tensile and shear crack distributions. Comparative studies have shown that as the confining pressure increases, the tensile cracks are restrained and inhibited and the splitting cracks along the loading direction gradually decrease and are replaced by more macroscopic shear cracks. Group C shows the fracture diagrams for the three sets of samples during the experiment and hand-drawn macroscopic fracture characteristics (black sketches). All three sets of experiments (C-1, C-2, C-3) show a transition from splitting cracks to shear zones as the confining pressure increases. In addition to this, an increase in the confining pressure generally leads to an enhancement in the intrinsic mechanical behaviour of the rock specimen (as shown in Table 5, Figure 19 and Figure 20), which includes: (a) an increase in peak breaking strength; (b) an increase in the initial crack stress threshold and damage stress threshold; (c) an increase in the microcrack count; (d) the dip angle of microcracks shifted from a concentrated axial direction of 90° to a more uniform distribution; (e) the damage mode of the rock gradually changes from brittle to ductile.

In summary, the patterns obtained from the numerical simulations are consistent with the experimental patterns. It further demonstrates that the UDEC-GBM model adopted in this study can effectively capture the key features of granite damage behavior.

## 5. Discussion and Conclusions

This paper investigates the damage mechanism and microcrack evolution signatures of granites with heterogeneous structures. The composition and contact heterogeneity of the rock microstructure is reconstructed in a numerical model using digital images. The intracrystalline fracture effect is constructed by forming multiple sub-polygonal blocks into crystalline particles of the same size as the actual one. The heterogeneity model was calibrated using triaxial and Brazilian splitting experiments, resulting in a numerical model that accurately reflects the macroscopic and microscopic dimensions.

(1) The fracture of microcracks occurs preferentially on the weak side of grain boundaries, and the appearance of intracrystalline shear cracks marks that the rock has reached its peak strength. The fracture of microcracks follows the following sequence of development: boundary-tensile crack, boundary-shear crack, transgranular-tensile crack, transgranular-shear crack, macro-shear bond. 

(2) Initial cracking occurs mainly in the vicinity of quartz. Transgranular breaks generally occur after crack damage stress threshold and are most numerous in quartz, followed by feldspar and least in biotite, which is consistent with the experimentally observed pattern and suggests that heterogeneous stress concentration-induced breaks are also an essential factor in inducing quartz breaks. Feldspars play an interconnecting role in developing microcracks and are more likely to form through cracks. Biotite has a lower modulus of elasticity and a higher Poisson’s ratio, which allows for more significant elastic deformation and is less prone to stress concentration, so microcracks usually surround or terminate at the biotite in the fracture path, with fewer intracrystalline cracks.

(3) An increase in confining pressure increases the number of boundary shear cracks, which gradually exceed the number of tensile cracks. The development of shear cracks at low confining pressures is slow until the peak strength and only increases dramatically at the post-peak. However, as the confining pressure increases, shear cracks grow sharply from the crack damage stress threshold and gradually suppress tensile cracks. The microcracks gradually transition from tensile to shear damage at the microscopic level. 

(4) At the macroscopic level, there is a shift from splitting cracks to shear zones, which is consistent with the regularity presented by the experiments. Under primary rock stress conditions, shear damage between the grains of the rock is the main factor causing macroscopic cracking. As the confining pressure increases, the peak strength increases and the total amount of cracks continues to increase, with more intracrystalline fracture after the peak stress and a higher risk of rock bursts. 

In this paper, the fractures between different crystal grains in microstructurally heterogeneous rocks are studied. It provides an explanation for the compression fracture mechanism in deep rock mass engineering. However, it is limited to two-dimensional exploration, and there is still a certain gap compared with the complexity of natural three-dimensional rock masses. Therefore, in future work, the study of three-dimensional fabric heterogeneity can be further considered.

## Figures and Tables

**Table 1:** Mineral composition of Biotite granite and simulation model.

Mineral	Composition (%)	
	Biotite Granite	Model
Plagioclase	36.2	31
K-feldspar	29.1	29
Quartz	27.8	24
Biotite	5.7	4.3
Kaolinite	0.2	/
Other	<1	/

**Table 2:** Mechanical properties of the minerals.

Mineral Type	Elastic Modulus (GPa)	Passion’s Ratio	Shear Modulus (GPa)	Bulk Modulus (GPa)	Density (Kg/m^3^)	Grain Size (mm)
Plagioclase	88.1	0.26	29.3	50.8	2630	2–5
K-feldspar	69.8	0.28	27.2	53.7	2560	2–5
Quartz	94.5	0.08	44.0	37.0	2650	2–5
Biotite	41.1	0.38	12.4	41.1	3050	0.5–3

**Table 3:** Corrected microscopic parameters.

Contact No	Contact Type	*k_n_* (N/m)	*k_s_*/*k_n_*	$c_{j}\;(\mathrm{MPa})$	$\varphi_{j\;,}\varphi_{jr}\;({}^{\circ})$	$t_{j}\;(\mathrm{MPa})$
1-1	Pl-Pl	9.28 × 10^13^	0.6	69	26.4	27
2-2	Kf-Kf	9.20 × 10^13^		68		25
3-3	Qz-Qz	2.55 × 10^14^		85		28
4-4	Bi-Bi	4.70 × 10^14^		50		23
1-2	Pl-Kf	9.24 × 10^13^		45		21
1-3	Pl-Qz	1.74 × 10^14^		45		21
1-4	Pl-Bi	2.81 × 10^14^		45		21
2-3	Kf-Qz	1.74 × 10^14^		45		21
2-4	Kf-Bi	2.81 × 10^14^		45		21
3-4	Qz-Bi	3.63 × 10^14^		45		21

$c_{j}$ joint cohesion, $\varphi_{j}$ joint friction angle, $\varphi_{jr}$ joint residual friction angle $t_{j}$ joint tensile strength.

**Table 4:** Error comparison between numerical model results and experimental results.

	Model	Lab	Error (%)
Peak Strength (MPa)	99.6	99.8	0.2
Young’s Modulus (GPa)	47.5	49.57	4.1
Passion’s ratio	0.23	0.24	4
Tensile strength (MPa)	8.9	9.52	6.5
Crack-Initiation Stress (MPa)	43	/	/
Crack Damage Stress(MPa)	89	/	/

**Table 5:** Analysis of numerical simulation results: Comparison of granite microcracks under different confining pressure conditions (0–20 MPa). Note: ‘Cs’ in crack statistics represents the occurrence of shear cracks.

Confine Pressure	Stress vs. Strain	Micro Cracks Count	Crack Orientation
0 MPa	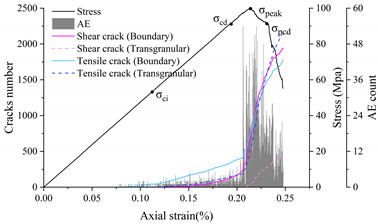	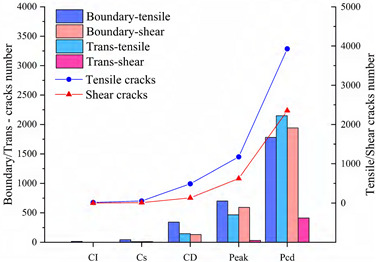	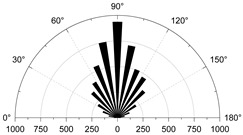
5 MPa	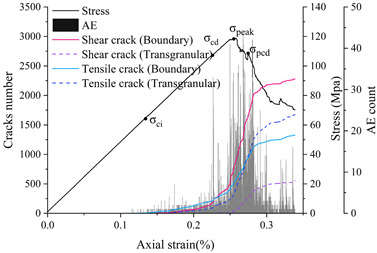	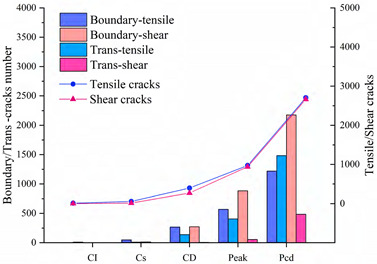	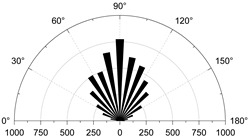
10 MPa	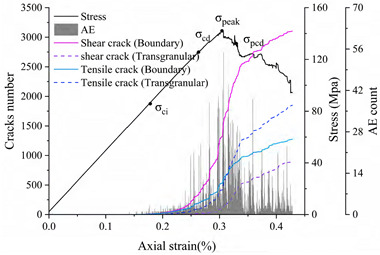	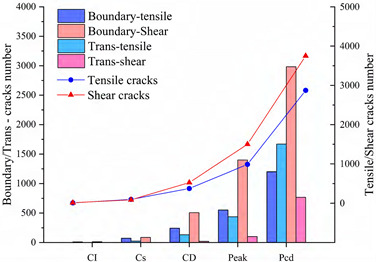	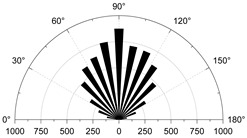
20 MPa	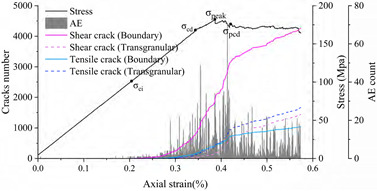	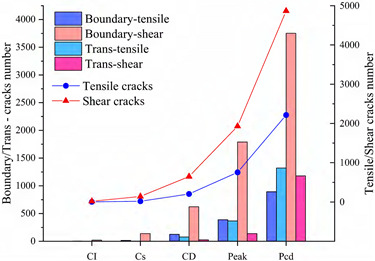	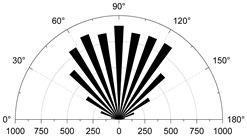

**Table 6:** Comparison of macroscopic rupture between numerical simulation and experiment under different confining pressure conditions. Note: Group A represents macroscopic fracture of numerical simulation; in Group B, blue and red colors represent tensile and shear cracks, respectively; Group C: 1–3, macroscopic fracture and sketch of test sample.

	0 MPa	5 MPa	10 MPa	20 MPa
A	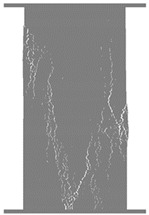	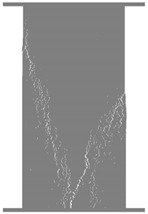	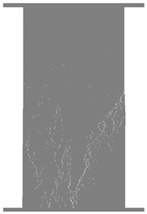	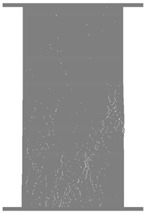
B	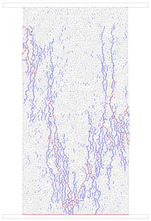	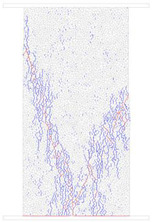	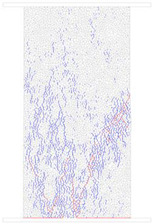	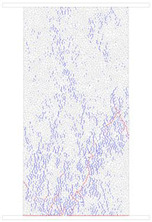
C-1	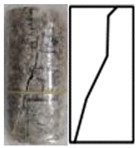	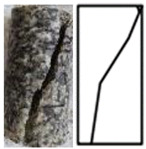	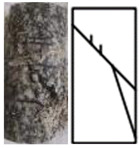	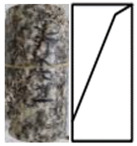
C-2	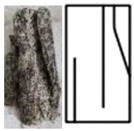	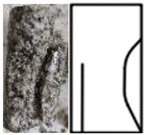	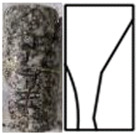	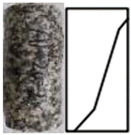
C-3